# Supplementary material for: Assembly and comparative analysis of the complete mitochondrial genome of three Macadamia species (M. integrifolia, M. ternifolia and M. tetraphylla)
Source: PLoS One. 2022 May 3;17(5):e0263545. doi: 10.1371/journal.pone.0263545 (PMC9064092; doi:10.1371/journal.pone.0263545)
Supplement: Attachment — Submitted filename: Response to Reviewers.doc [file pone.0263545.s006.doc]

**Reviewer #1**: Overall, the manuscript is well written and data/findings are clearly presented. The methods can be expanded and I have made notes on the manuscript to direct the authors on where to enhance the methods. Likewise, I have provided suggestions for additional literature that can be included in the manuscript at the discretion of the authors.

Thank you very much for your guidance. We have made necessary modifications according to the marks.

**Reviewer** #2: The authors presented a study on three mitogenomes from Macadamia species, and did the comparison of genomic features, repeat sequences, RNA editing, transfer from plastome into mitogenome and phylogeny with other higher plants. This manuscript provides some interesting findings. But there are still a few questions that need to be clarified and improved. In order to make the manuscript much clearer and the conclusions more valid, comments as follows:

**1. The Figure 1 on the circular map of three *Macadamia* mitogenomes, the authors should remake these maps, clear labels for each gene.**

The Figure 1 had been redrawn and uploaded in the new manuscript.

**2. I suggest the authors take care of the space and consistence on the number and word, for example, “31841bp (cox2)”, there is no space between “31841” and “bp”, but in other places there is space between them; also for the number, “31841” using “31,841” style, etc.**

We checked the full text and corrected similar errors.

**3. The other big problem is the references, the author must be very careful on every reference, such as uppercase, lowercase, italic, journal names, etc. The author must follow the instructions of the reference of this journal.**

All references have been corrected to Plos ONE style.

**4. What are the structural differences among the three mitogenomes? How the author verify the accuracy of the three assemblies, especially for a couple of bps difference among these genomes?**

As mentioned in the material method, depth of coverage was used to correct mitochondrial sequence information. SPAdes v.3.5.0 software was used to splice and assemble mt genome sequences. To correct the splicing results, the raw sequencing data were mapped to mitochondrial sequences using Geneious software.

**5. In the part of “Up to 34.3% (236 sites) RNA editing sites occurred in the first base position of the codon, 68.6% (472 sites) appeared in the second base position, and there was no RNA editing in the third base position.”, why the total percentage > 100%?**

This statement has been modified in the new manuscript. 236 RNA editing sites occurred in the first base position of the codon, 472 sites appeared in the second base position, and there was no RNA editing in the third base position.

**6. For the Phylogenetic analysis, the authors need to provide more interesting information or findings.
7. I suggest the authors add more findings from the structural and evolution innovations from these mitogenomes, such as group I and II** **introns, etc.**

We have revised the above two points in the new manuscript. Thank you very much for your guidance
